# Biodegradable Antioxidant Composites with Almond Skin Powder

**DOI:** 10.3390/polym17162201

**Published:** 2025-08-12

**Authors:** Irene Gil-Guillén, Idalina Gonçalves, Paula Ferreira, Chelo González-Martínez, Amparo Chiralt

**Affiliations:** 1 Institute of Food Engineering-FoodUPV, Universitat Politècnica de València, 46022 Valencia, Spain; cgonza@tal.upv.es; 2 Department of Materials and Ceramic Engineering, CICECO–Aveiro Institute of Materials, University of Aveiro, 3810-193 Aveiro, Portugal; idalina@ua.pt (I.G.);

**Keywords:** almond skin, composite films, PLA, PVA, antioxidant properties

## Abstract

Almond skin (AS) from industrial almond peeling is considered an agri-food waste with adequate composition to obtain composite films for food packaging due to its richness in polysaccharides, proteins, and phenolic compounds. Composite films based on amorphous polylactic acid (PLA) or partially acetylated polyvinilalcohol (PVA) were obtained by melt blending and compression moulding, incorporating different ratios of defatted AS powder (0, 5, 10, and 15 wt.%). The filler was better integrated in the polar PVA matrix, where more interactions were detected with the filler compounds, affecting glass transition and crystallization of the polymer. The AS particles provided the films with the characteristic colour of the powder and strong UV light-blocking effect, while improving the oxygen barrier capacity of both polymeric matrices (24% in PLA with 15% AS and 42% in PVA with 10% AS). The water vapour permeability increased in PLA (by 192% at 15% AS), but decreased in PVA films, especially with low AS content (by 19% with 5% particles). The filler also provided the PLA and PVA films with antioxidant properties due to its phenolic richness, improving the oxygen barrier capacity of the materials and delaying the unsaturated oil oxidation. This was reflected in the lower peroxide and conjugated dienes and trienes values of the sunflower oil packaged in single-dose bags of the different materials. The high oxygen barrier capacity of the PVA bags mainly controlled the preservation of the oil, which made the effect of the antioxidant AS powder less noticeable.

## 1. Introduction

Food packaging is an essential aspect for maintaining the safety, integrity, and quality of food, as well as extending its shelf life during transportation and storage processes [1,2]. Polypropylene (PP), polyamide (PA), polyethylene terephthalate (PET), and polystyrene (PS) are the most used polymers in these preservation systems [3,4]. Nevertheless, the massive use of non-biodegradable plastics has caused serious environmental problems due to the accumulation of plastics waste [5,6]. To address this problem, various strategies must be applied, ranging from conscious consumption and material recycling to the generation of compounds that can biodegrade in composting spaces or in different ecosystems. Numerous studies using biodegradable polymers have been conducted to develop biodegradable packaging materials that ensure food safety and quality while minimising harmful environmental effects [7,8,9].

Of the biodegradable/compostable polymers, polylactic acid (PLA) and polyvinilalcohol (PVA) offer interesting properties for food packaging applications, being a more sustainable alternative that could reduce plastics waste. PVA has great potential in this area due to its high oxygen barrier capacity, transparency, colourlessness, and non-toxic properties. However, due to its high water vapor permeability, it is not recommended for use in foods with high water content [10,11]. In contrast, PLA has excellent moisture barrier properties and high mechanical strength, characteristics that make it an optimal polymer for wet food packaging applications [12,13]. Nonetheless, due to its rigid structure, it shows brittleness when exposed to tensile and bending forces [14]. Multilayer films of both polymers have been proposed to produce laminates with adequate barrier capacity for food packaging; PVA provides the laminates with high barrier to oxygen, whereas PLA provides an effective barrier to water vapour [11].

The high cost of the biodegradable polymers could be reduced for food packaging applications by incorporating lignocellulosic particles of agri-food waste, which, in turn, could provide the obtained biocomposites with bioactive properties (antioxidant/antimicrobial) from the natural phenols present in the waste [15,16]. The biocomposites may improve the quality and safety of packaged food, especially in products that are highly susceptible to oxidation [17]. Packaging solutions containing active ingredients can prolong the food shelf life, extending the distribution time in the market while reducing the plastic waste generated [18,19].

Many studies have been carried out to obtain biocomposites with polymeric materials by incorporating different agri-food wastes [20,21]. Almonds are highly produced worldwide (4.6 Mt in 2021) [22], and an important part of their production is industrial peeling via blanching, generating almond skin waste with a high yield, since it represents 6–8% of the total weight of the fruit. It is often used for animal feeding or biofuel obtention [23,24]. However, this waste has an interesting composition for use in higher-value applications. It contains a large amount of dietary fibre and a wide range of bioactive phenolic compounds [25,26], such as hydrolysed tannins, phenolic acids, aldehydes (11.6 mg/100 g), flavonoids (71.3 mg/100 g), and proanthocyanidins (6.98 mg/100 g) [27]. Numerous functional properties of these phenolic compounds have been demonstrated, including the ability to activate antioxidant enzymes and prevent the activation of enzymes that initiate lipid oxidation processes, neutralise free radicals, and chelate metals [28,29]. Fractionation of the almond peel into fibres and phenolic-rich extracts with antioxidant and antimicrobial properties have been previously studied by applying subcritical water extraction [23]. Likewise, their use as a powder filler within different polymer matrices, such as PCL [30,31], PLA [32,33], or polyamide6 [34], has been studied for different applications.

Given the high content of fibre and antioxidant/antimicrobial compounds of AS [23], incorporating this powdered residue into different polymer matrices could be an interesting strategy for developing active composite materials for food packaging. The fibres could modulate the barrier and mechanical properties of the polymer matrix, and the active compounds could be released into the food substrate, thus extending its shelf life. Therefore, it is necessary to study the effect of AS powder on the functional properties of polymeric materials to demonstrate their suitability for food packaging applications. In this regard, a different impact of AS filler would be expected depending on the nature of the polymer (hydrophilic or hydrophobic) and the interactions of the AS particles/compounds with the polymer chains. These would affect the barrier and mechanical performance, as well as the antioxidant or antimicrobial capacity, which are crucial in food packaging requirements.

The aim of this study was to produce PVA and PLA composite films with defatted almond skin (AS) powder to lower the cost of food packaging materials while providing the films with antioxidant capacity. The biocomposite films were obtained by melt blending and compression moulding with different ratios of AS (0, 5%, 10%, and 15%) and characterised as to their functional (mechanical, barrier, and optical) properties as packaging materials as well as to their microstructure and thermal behaviour. Likewise, their antioxidant capacity and their ability to prevent the oxidation of sunflower oil (as an unsaturated fat model) were analysed.

## 2. Materials and Methods

### 2.1. Materials

Amorphous PLA 4060D was supplied by NatureWorks (Plymouth, MN, USA), with a molecular weight of 106 kDa. PVA, with a molecular weight of 30–70 kDa and a hydrolysis of 87–90%, was purchased from Sigma-Aldrich (Steinheim, Germany). Other reactants including P_2_O_5_, Mg (NO_3_)_2,_ isooctane, glacial acetic acid, 1-decanol, potassium iodide, and potassium permanganate pentahydrate were also supplied by Sigma-Aldrich (Steinheim, Germany). Importaco S.A. (Valencia, Spain), who produce peeled almonds, generously provided almond skin (AS) samples (Prunus dulcis, var. California Nonpareil). Commercial sunflower oil (Consum, Valencia, Spain) with 11% saturated, 30% monounsaturated, and 59% polyunsaturated fats was used to the antioxidant test.

### 2.2. Obtaining Almond Skin (AS) Powder

The plant material was dried at 40 ± 2 °C for three days in a forced-air oven (S. P. Selecta, s.a., Barcelona, Spain). It was then milled using a grinding machine (IKA, model M20, Staufen, Germany) and subjected to a degreasing process with petroleum ether (40–60 °C) for six hours by Soxhlet extraction. The defatted powder was sieved to obtain particles with a size lower than 63 µm, which were used to prepare the composite films. The powder was stored at 0% relative humidity (HR) (in desiccator with P_2_O_5_) to prevent wetting. The composition of the defatted almond skin (AS) powder was reported in a previous study [23], containing 13% protein, 10% cellulose, 12% hemicellulose, 17% lignin, and about 3% total phenol content, while its aqueous extracts exhibited radical scavenging capacity (5 mg. mg^−1^ DPPH).

### 2.3. Obtaining the Films

To produce PLA and PVA films, a melt blending process was carried out with different proportions of AS powder (0, 5, 10, and 15 wt.%). In PVA films, glycerol was used as a plasticiser at 10 wt.% in the films. Table 1 show the mass fraction of each component in the composite films. The different components of each film formulation were previously mixed in powder and then melt blended by using an internal mixer (HAAKETM PolyLabTM QC, Thermo Fisher Scientific, Karlsruhe, Germany). Subsequently, the mixtures were cold-milled with liquid N_2_ to obtain powder that was thermoformed into films (4 g per film) with a hydraulic press (model LP20, Labtech Engineering, Samut Prakan, Thailand). The amorphous PLA pellets were previously dried and conditioned in P_2_O_5_ for two days to remove moisture.

The PLA blends were melt-blended at 160 °C and 50 rpm for 6 min. Thermoforming of the films included preheating at 160 °C for 3 min, followed by compression at 100 bar and 160 °C for 3 min, and a final cooling to 80 °C for 3 min. The PVA blends were obtained at 180 °C and 50 rpm for 10 min. Thermoforming involved preheating at 180 °C for 3 min, a 3 min compression at 100 bar and 180 °C, and a final cooling to 80 °C in 3 min. The temperatures were selected based on the melting point of each polymer.

The obtained films (Table 1) was conditioned at 0% RH or 53% RH, depending on the test to which they were submitted.

### 2.4. Characterisation of the Films

#### 2.4.1. Oxygen and Water Vapour Barrier Properties

The water vapour permeability (WVP) of the films conditioned at 53% RH was determined gravimetrically, following the ASTM E96/E96M standard [35]. Circular samples (3.5 cm) sealed in Payne permeability cups with 5 mL of distilled water (100% RH) were placed in desiccators at 25 °C with oversaturated Mg(NO_3_)_2_ solution (53% relative humidity: RH), generating a constant RH gradient (100%–53% RH). The cup weight was recorded every 75 min for 27 h with an analytical balance. The water vapour transmission rate (WVTR) was obtained from the slope of the weigh vs. time curve once the steady state was reached. WVP was calculated from the WVTR, considering the film thickness (measured with a digital micrometre (Palmer, COMECTA, Barcelona, Spain) and the vapour pressure gradient. The analysis was performed in triplicate for each formulation.

The oxygen permeability (OP) of the films conditioned at 53% RH was evaluated with oxygen permeation equipment, Model 8101e equipment (Systech Illinois, Plymouth, MN, USA), according to ASTM D3985-05 [36]. The oxygen transmission rate (OTR) through 50 cm^2^ samples was recorded every 15 min until equilibrium was reached. Measurements were taken in triplicate for each sample. The OP was calculated by multiplying the OTR by the film thickness and dividing by the oxygen partial pressure gradient.

#### 2.4.2. Optical Properties

The reflection spectrum of the films conditioned at 53% RH was measured between 400 and 700 nm with a spectrocolourimeter (CM-3600d, Minolta, Tokyo, Japan), using a white background with known reflectance (Rg) and a black background (R_0_). Based on these data and applying the Kubelka–Munk theory (Equations (1)–(3)), the reflectance at infinite thickness (R_∞_) was calculated. Using R_∞_, the CIE L*a*b* colour coordinates (illuminant D65, 10° observer) were obtained, and from a* and b*, the hue (h_ab_*) and chroma (C_ab_*) were derived (Equations (4) and (5)). Finally, the total colour difference (ΔE*) between films with AS and the respective control was calculated (Equation (6)).

(1) $$R_{\infty }=a-b$$

(2) $$a=\frac{1}{2}\left[R+\left(\frac{R_{o}-R+R_{g}}{R_{o}\cdot R_{g}}\right)\right]$$

(3) $$b=\sqrt{a^{2}-1}$$

(4) $$h_{ab}*=arctg(\frac{b*}{a*})$$

(5) $$C_{ab}*=\sqrt{{a*}^{2}+{b*}^{2}}$$

(6) $$∆E*=\sqrt{{\left(∆L*\right)}^{2}+{\left(∆a*\right)}^{2}+{\left(∆b*\right)}^{2}}$$

#### 2.4.3. UV-Protection Capacity

The UV-protective capacity of the films conditioned at 53% RH was monitored through the UV-visible spectra, using a spectrophotometer, model UV-3600 i Plus (Shimadzu, Tokyo, Japan) from 200 to 800 nm. For each spectrum, a representative average of 3 scans was obtained using a data pitch of 0.5 nm, a bandwidth of 2.0 nm, and a scanning speed of 100 nm/min.

#### 2.4.4. Mechanical Properties

Tensile tests were carried out in the films conditioned at 53% RH, using a universal press (TA.XTplus, Stable Micro Systems, Haslemere, UK), applying the ASTM D882 standard [37]. The force–deformation curves of the films were transformed to Hencky stress–strain curves and the elastic modulus (EM), derived from the initial slope of the curve, as well as the tensile strength (TS) and the percentage strain at the breaking point (E%) were determined. The film specimens were prepared (25 × 100 mm), placed in the grips 50 mm apart, and pulled at a rate of 50 mm/min. Each formulation was evaluated with six samples.

#### 2.4.5. Wettability

Static water contact angles (WCA) with the different films conditioned at 53% RH were measured at room temperature using a Biolin Scientific Attension Theta equipped with a OneAttension imaging system (Paralab, Valbom, Portugal), as described by Peixoto et al. [38]. WCAs were calculated following the Laplace–Young method, and four measurements were performed in each film.

#### 2.4.6. Fourier Transform Infrared Spectroscopy (FTIR)

The IR spectra of the films conditioned at 53% RH were obtained using an FTIR spectrometer, model Alpha II (Bruker, Santa Clara, CA, USA). The absorbance spectra were obtained using an average of 128 scans with a resolution of 4 cm^−1^, in the wavelength range of 400 to 4000 cm^−1^. The film samples were cut into squares (2 × 2 cm), and the measurement was carried out in triplicate.

#### 2.4.7. Microstructural Analysis

The microstructure of the films conditioned at 0% RH (P_2_O_5_) was analysed by FESEM (Ultra 55, Zeiss, Oxford Instruments, Oxford, UK). The film samples were cryofractured in slush nitrogen to expose their cross-section, then fixed in holders, coated with platinum, and analysed at 1.5 kV under the microscope.

#### 2.4.8. Thermal Analysis

A thermogravimetric analysis (TGA) was conducted to evaluate the thermal stability of the films conditioned at 0% RH, using a TGA 1 STARe System (Mettler-Toledo Inc., Greifensee, Switzerland). Between 3 and 5 mg of the sample were weighed, placed in an alumina crucible, and subjected to heating from 25 °C to 600 °C at a rate of 10 °C/min, under a nitrogen atmosphere with a flow rate of 10 mL/min. The STARe software (version V12.00a, Mettler-Toledo, Greifensee, Switzerland) generated the DTGA curves, which were used to establish both the initial temperature and the maximum degradation rate temperature of each moss loss step. Each analysis was carried out in duplicate on films conditioned to 0% relative humidity (RH).

The DSC analysis was carried out using a differential scanning calorimeter (STARe System, Mettler-Toledo Inc., Greifensee, Switzerland). Film samples weighing 3–5 mg were placed in aluminium pans and submitted to consecutive heating, cooling, and second heating steps, under a constant nitrogen flow (30 mL/min), using an empty pan as reference. The PVA samples were heated from 20 °C to 210 °C at 10 °C/min, followed by a 1 min hold at 210 °C, then cooled to 20 °C at 10 °C/min with a 1 min hold, and finally reheated to 210 °C at 10 °C/min. The PLA samples were heated from 25 °C to 160 °C at 10 °C/min, holding for 1 min at 160 °C, cooled to 25 °C at 10 °C/min, and reheated to 160 °C at 10 °C/min. The tests were conducted in duplicate for each film formulation conditioned at 0% relative humidity (RH). From the second heating stage, the glass transition temperature (Tg), melting temperature (Tm), and melting enthalpy (ΔHm) were determined, while the crystallisation temperature (Tc) was obtained from the cooling step.

#### 2.4.9. Dynamic Mechanical Analyses (DMA)

Dynamic mechanical analysis (DMA) of the films conditioned at 53% RH was carried out using a Tritec 2000 equipment (Triton Technologies, Keyworth, UK). Film samples (3 cm × 0.5 cm) were subjected to dynamic compression deformation amplitude 0.1%, frequency 1 Hz) from 20 °C to 120 °C at a constant heating rate of 3 °C/min.

#### 2.4.10. Antioxidant Capacity of the Films

The antioxidant activity of the film was determined through their ability to inactivate the ABTS^+^ radical (2,2′-azino-bis(3-ethylbenzothiazoline-6-sulfonic acid)) and their capacity to prevent sunflower oil oxidation when packaged in bags of the different films.

##### Inhibition Capacity of ABTS^+^

To determine the film’s capacity to inhibit the ABTS^+^ (2,2′-azino-bis-(3-ethylbenzothiazoline-6-sulfonic acid) radical, an adaptation of the method described by Nunes et al. [39] was used. Briefly, the ABTS^+^ was produced by reacting ABTS (7 mM) with potassium persulphate (2.45 mM) in the dark, at room temperature, for 16 h. The ABTS^+^ solution (1 mL) was diluted in ethanol (about 80 mL) to achieve an absorbance between 0.700 and 0.800 at 734 nm, measured with a spectrophotometer (Jenway 6405 UV/Vis, Stone, Staffordshire, UK). Film samples (1 cm^2^) were immersed in 1.5 mL of the ABTS^+^ solution and left to react under dark conditions at room temperature with orbital stirring (80 rpm), while absorbance at 734 nm was measured after different reaction times (Af). ABTS^+^ solution without film was used as a blank. Absorbance at 734 nm of the solutions was measured in triplicate overtime for 72 h. The antioxidant activity was determined by the ABTS^+^ inhibition percentage, calculated by applying Equation (7), at each contact time. The assay was performed in triplicate for each film.

(7) $$Inhibition\;\left(\%\right)=100\times \frac{Ab-Af}{Ab}$$

where A_b_ and A_f_ correspond to the absorbance values of the ABTS^+^ solution incubated without and with the film, respectively.

##### Prevention of Sunflower Oil Oxidation

The antioxidant capacity of the films was also evaluated by an accelerated oxidation test in packaged sunflower oil. For this purpose, single-dose bags (100 × 50 mm) heat-sealed with a vacuum packer (Vacuum Press, Saeco, Jinan, China) were prepared with the different films, filled with 5 mL of oil, and sealed. The samples were stored at 40 °C, 53% RH, and fluorescent illumination (1000–1500 lx) for 45 days. The oil samples were analysed at baseline and every 15 days to monitor oxidation progress through the control of peroxide values and conjugated dienes and trienes. Oil samples from two bags per material were analysed at each time.

The peroxide value was determined according to EU Regulation No 2568/91 [40] by dissolving 1 g of oil in 10 mL of a 3:1 mixture of glacial acetic acid and decanol. Potassium iodide (200 µL) was added, allowed to stand for one min in darkness, and titrated with Na_2_S_2_O_3_ (0.01 or 0.001 M) using an automatic titrator (Titrando, Metrohm, Herisau, Switzerland).

For conjugated dienes and trienes, absorbance was measured at 232 nm and 268 nm, respectively, according to EU Regulation No 2568/91 [40]. Oil samples (0.12 g) were dissolved in 50 mL isooctane and the UV measurements were performed. The results were expressed as absorption coefficients (Equation (8)).

(8) $$K\lambda =\frac{E\lambda }{c*s}$$

where Kλ is the extinction coefficient at a given wavelength, Eλ is the absorbance measurement, c is the oil concentration in g/100 mL, and s is the thickness of the quartz cuvette used in cm.

### 2.5. Statistical Analysis

The data was statistically analysed using analysis of variance (ANOVA) with Statgraphics Centurion XVII-X64 (StatgraphicsTechnologies, Inc., Rockville, MD, USA), employing Fisher’s Least Significant Difference at the 95% confidence level.

## 3. Results and Discussion

### 3.1. Molecular and Structural Properties of the Films

**Figure 1:**
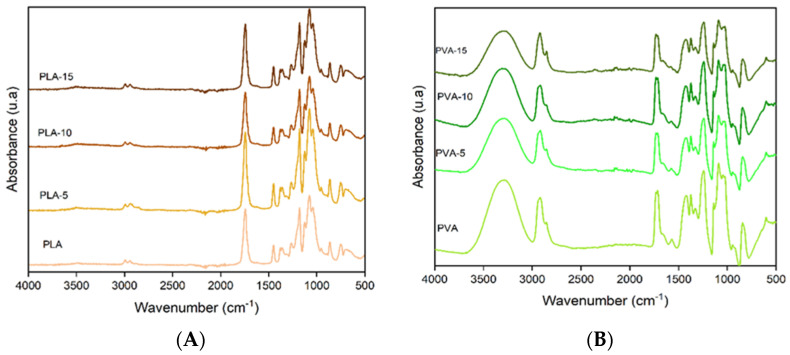
FTIR spectra of PLA (**A**) and PVA (**B**) films with 0%, 5%, 10%, and 15% of almond skin powder.

Figure 1 shows the Fourier transform infrared (FTIR) spectra associated with the different PLA and PVA films, with different percentages of almond skin powder (0, 5, 10, and 15%). Spectral analysis makes possible the evaluation of possible interactions between the components of the mixtures, as well as the recognition of the distinctive functional groups found in each formulation. In PLA films, characteristic bands belonging to the stretching vibration of the carbonyl group (C=O) can be observed at 1740 cm^−1^, while bands associated with the vibration of C-O bonds appeared at 1180–1080 cm^−1^ [41,42]. The peaks observed at 2995 and 2945 cm^−1^ corresponded to the asymmetric and symmetric stretching vibrations of CH_3_, respectively. Additionally, the band at 1455 cm^−1^ was associated with the asymmetric bending vibration of CH_3_ [43]. No significant changes in the FTIR spectra of the PLA films were observed when AS powder was incorporated into the matrix at different ratios. This can be attributed to the relatively low proportion of the different AS components in the film and the lack of remarkable interactions between these compounds and the polymer. Karagöz et al. [44] only observed significant changes in the FTIR spectra of PLA composites with a walnut shell filler from 30% of filler, when the hydroxyl vibration band of the fibres exhibited a significant intensity.

In the case of PVA films, a broad band in the 3200–3400 cm^−1^ region, corresponding to the O-H stretching typical of polyvinyl alcohol, was observed. Likewise, the bands observed at 2900 cm^−1^ and 1090–1140 cm^−1^ can be attributed to the vibration of the C-H and C-O bonds, respectively [45]. The two bands at 2910 cm^−1^ and 2858 cm^−1^ correspond to the asymmetric and symmetric stretching vibrations of C-H and of -CH_2_ groups, and the peaks in the region 1418–1236 cm^−1^ were attributed to the bending vibrations pf C-H and O-H [46]. At 1713 and 1730 cm^−1^, the double peak characteristic band C=O vibration of the residual acetate was observed. The splitting of the carbonyl peak has been attributed to the presence of hydrogen-bonded carbonyls (at the lowest wavenumber, 1713 cm^−1^) and non-hydrogen-bonded C=0 (at the highest wavenumber, 1730 cm^−1^) [47]. The changes in the intensity ratio of both bands (I_1713_/I_1730_) may indicate different degrees of hydrogen bonds in the polymer matrix [48]. Incorporation of AS powder in PVA composites produced a small reduction in this ratio (from 1.09 in PVA film to 0.98 in PVA-15 film), suggesting a slight decrease in the bonded carbonyls in the matrix. Likewise, the ratio between the intensity of band at 2910 cm^−1^ (C-H vibration) and at 3357 (O-H vibration) (I_2910_/I_3357_) rose as the AS content increase in the composite (from 0.65 in PVA films to 0.97 in the PVA-15 films). These changes suggest that new hydrogen bonds between the PVA hydroxyls and AS compounds containing hydroxyls, such as phenols, lignin or carbohydrates, could be formed in the composite matrices, as reported by Barbălată-Mândru et al. [46] for PVA films with different plant extracts. These new hydrogen bonds could interfere with and limit the interchain bonds usually present in the PVA matrix, affecting the film properties.

**Figure 2:**
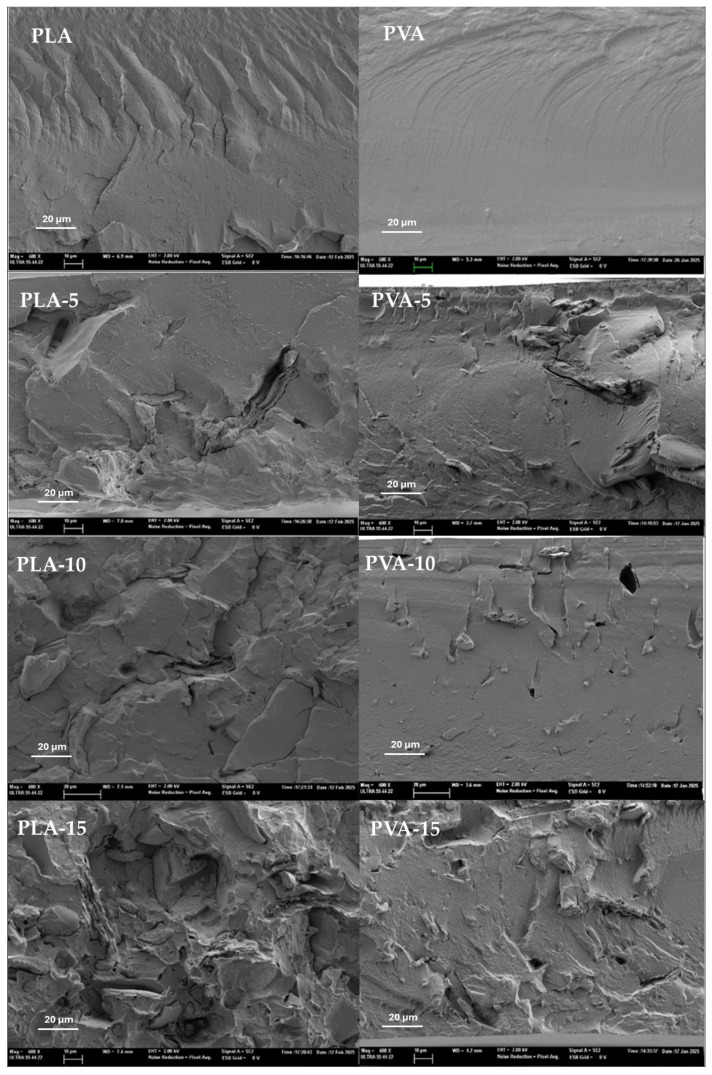
FESEM images (×600) of the PLA (**left**) and PVA (**right**) composite films with different ratios (0%, 5%, 10%, and 15%) of AS powder.

**Figure 3:**
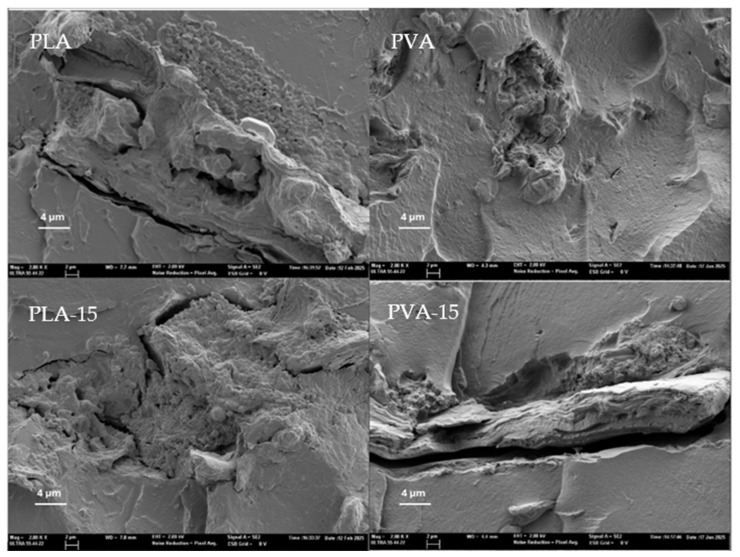
FESEM images (×2000) of AS particles embedded in PLA (**left**) and PVA matrices (**right**).

Figure 2 and Figure 3 show the FESEM images of the film cross-section for the different PLA and PVA composite films, in comparison to the control films without AS. In composites, a different ratio of dispersed particles can be observed embedded within the polymer matrix. Most of these particles exhibited a fibrous morphology, corresponding to the lignocellulosic fragments of the plant material. However, aggregates of small spherical particles were also observed at higher magnification (Figure 3), which can be attributed to the protein bodies originating in the AS with 13% protein.

The pure PLA and PVA films showed the characteristic morphology previously described in other studies [49,50], but incorporation of AS powder in the composites led to a more heterogenous structure. Components of AS particles could be partially miscible with the polymer chains; some molecular compounds can migrate to the polymer matrix during melt blending, depending on the chemical affinity, whereas other components, such as cellulose fibres, will remain dispersed into the polymer continuous matrix. The cryofractured surface of PLA films with AS powder showed the dispersed particles or the void left by them during cryofracture. This effect was most noticeable in the sample PLA-15 with 15% AS, where the heterogeneity degree in the film is more remarkable and the continuity of the polymer matrix was highly reduced. In contrast, the PVA films showed a better integration of the AS particles and, even with 15% AS, the continuous PVA matrix was clearly defined, showing a good adhesion of the powder particles and the polymer, as no remarkable gabs were detected around the particles (Figure 3). Similar behaviour was observed for PVA composite films with cellulose fibres from almond shell, where the fibres appeared to be partially covered by a compact polymeric layer firmly adhered to their porous surface [50].

The poor integration of AS particles in the PLA matrix can be attributed to the hydrophobic characteristics of PLA, whereas almond skin particles are rich in hydrophilic compounds, such as polysaccharides (cellulose hemicellulose), lignin, proteins, and phenols [23], with an inherent incompatibility with the PLA matrix. However, the particles appeared to be well dispersed in the matrix without agglomeration. Similar structural effects were observed in other PLA composite films with untreated walnut shell [44].

### 3.2. Optical and UV Protection Properties

**Figure 4:**
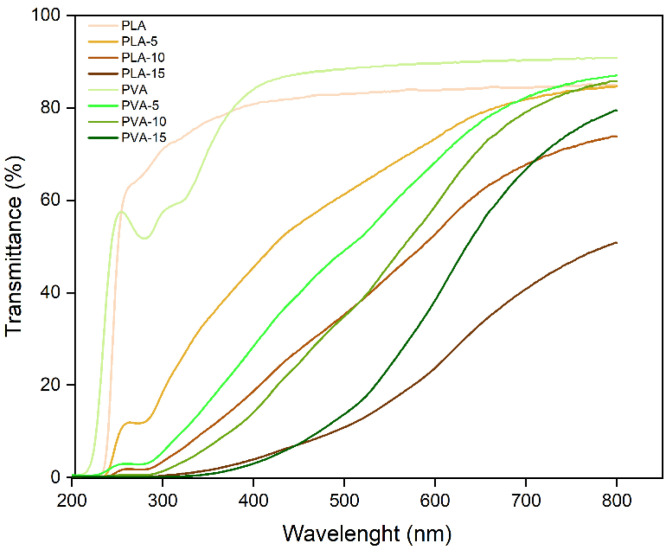
Internal transmittance spectra in the visible range of different PLA and PVA composite films with 0, 5, 10, and 15 wt.% of almond skin powder.

Incorporation of AS powder into the polymer matrices resulted in a significant decrease in the film lightness, and an increase in colour saturation, while hue veers to redder, according to the typical colour of almond skin powder. These changes intensified with the increase in AS percentage, thought differently for both polymers. It is remarkable that PVA composite films became darker, redder, and less saturated in colour than the corresponding PLA composites, as shown in Table 2. This could be due to a release of more coloured compounds from the AS particles to the PVA matrix in line with the higher temperature of processing (180 °C vs. 160 °C in PLA) and the higher polarity of this polymer with greater chemical affinity with these compounds. This was also reflected in the light transmission spectra of the films (Figure 4).

The incorporation of different percentages of AS particles produced a significant decrease in the film transparency, especially in the low-wavelength region (Figure 4). The increase in the particle ratios in the matrix promoted light scattering, as well as light absorption by the chromophore groups of the AS compounds, resulting in a progressive decrease in the internal transmittance as the AS ratio rose. However, as observed in the colour coordinates, a different effect was produced in PLA and PVA matrices at a determined AS content. The increase in opacity due to the particles was much more pronounced in the PVA films than in the PLA films, especially at low wavelengths. This can be attributed in part to the different refractive index of the polymers but also to the different release of coloured compounds from the AS particles into the polymer matrix during thermoprocessing, as commented on above. The higher polarity and chemical affinity of PVA for the coloured compounds present in the almond skin could enhance their release with the consequent higher pigmenting effect on the film. This light blocking effect was extended at the UV region due to the UV light absorption of phenols present in the AS particles. Therefore, the composite films exhibited a high UV light blocking effect, which represents a great advantage for inhibiting UV-induced oxidation reactions in packaged food sensitive to oxidation.

### 3.3. Mechanical and Barrier Properties of the Films

Molecular interactions and structural modifications caused by the incorporation of almond skin powder in PVA and PLA matrices caused changes in the mechanical and barrier characteristics of these films, which are shown in Table 3. Table 4 shows the film thickness and the equilibrium moisture content of the films, which may also affect mechanical response. Specifically, moisture content greatly affects the properties of PVA films due to their hydrophilic nature and moisture sensitivity [51].

The tensile parameters of the PLA films were in the range previously reported by other authors for the same type of amorphous polymer [52]. Incorporation of the AS powder did not practically affect the elastic modulus of the material but tended to decrease the resistance to breaking and extensibility of the films as the AS ratio rose. This can be attributed to the decrease in the chain interaction forces in the polymer matrix, generated by the particle interruption of chain aggregations. Therefore, AS particles did not produce a reinforcing effect in the PLA matrix, as reported by other authors for other cellulosic particles within PLA films [53,54]. The complex composition of the particles, with high levels of lignin and phenolic compounds [23], probably made their reinforcing effect difficult in the hydrophobic PLA matrix. However, only a 32% reduction in the resistance to fracture at the highest powder concentration (15%) was detected, which does did not imply a remarkable decrease in mechanical performance.

Tensile proprieties of PVA are greatly affected by different factors, such as the molecular weight and hydrolysis degree of the polymer, the ratio of plasticiser (usually incorporating glycerol), and the moisture content of the films [51]. Likewise, the film processing method also affects the properties of the films [10]. The values of obtained tensile parameters of neat PVA films were in the range previously reported by other authors for films of PVA with similar molecular weight (Mw), hydrolysis degree, glycerol content, and processing conditions [10,50]. Incorporation of AS powder at 5% level into the less-stiff PVA films provoked a 30% decrease in the elastic modulus that tended to be reduced when the AS ratio rose. Likewise, resistance to break and stretchability were progressively reduced as the AS ratio rose, reaching 52% and 32% reductions, respectively, at 15% AS. Therefore, the PVA matrix interruption with the AS particles also promoted its weakening, despite their apparently better integration within the polymer matrix. However, not only the dispersed particles but the molecular compounds potentially released into the PVA matrix may affect its mechanical performance by interacting with the polymer chains and reducing the interchain bonds within the matrix. Moreover, mechanical properties of PVA are greatly affected by the moisture content since water molecules highly plasticise the polymer, reducing the interchain forces. The incorporation of AS tended to slightly reduce the equilibrium moisture content of the films (Table 4) but slightly promoted their wetting capacity since the contact angle decreased from 44° in PVA films to 35–39° in composites. This suggests that the AS particles could modify the water relations within the polymer matrix, thus also affecting mechanical behaviour. Likewise, glycerol can interact with both polymer and filler, producing a component partition between phases and modifying the plasticising effect in the polymer matrix. In contrast, the equilibrium moisture of PLA was very low, as expected from its hydrophobic nature, and it was practically unaltered by the AS filler at different ratios. Nevertheless, the wetting properties of these films were promoted by the AS particles since the contact angle decreased from 92° to 64°, as observed by Li et al. [55] for PLA composite films with hydrophilic fillers such as chitosan.

Water vapor and oxygen permeability values of the different films are also shown in Table 3. Films of neat polymers exhibited values in the range previously reported by other authors [11,50,52,56], with the PLA being more permeable to oxygen and less permeable to water vapor than PVA, according to their respective hydrophobic and hydrophilic natures. Incorporation of AS powder in both polymers increased the oxygen barrier capacity of these materials (24% for PLA with 15% AS and 42% for PVA with 10% powder, with no further increase for 15% AS). In contrast, AS powder promoted the WVP of PLA films (192% at 15%) while reducing the values of PVA (18% for 5% AS). Nevertheless, this reduction decreased when the AS ratio rose (only 8% reduction for 15% AS).

The effect of the almond skin particles may be explained in terms of the chemical affinity and diffusion coefficient for oxygen and water molecules of the particles with respect to the polymer. The dispersed particles could imply an impediment for mass transfer when these have slower mass transfer rate than the polymer matrix, or the acceleration of the global transport when it is faster through the particles. The fat-free AS particles, rich in polysaccharides, lignin, and phenolic compounds [23], with crystalline fractions of cellulose, would be less permeable to oxygen than both PLA and PVA matrices and more permeable to water molecules than PLA but potentially less permeable than PVA. Therefore, the particles enhance the oxygen barrier capacity of the films but accelerate the water transfer rate in PLA films while introducing more obstacles for water transfer in PVA films. Likewise, the presence of antioxidant compounds in the AS particles, potentially diffused in the polymer matrix, can function as oxygen scavengers, reducing OP [56].

### 3.4. Glass Transition and Crystallisation Behaviour

**Figure 5:**
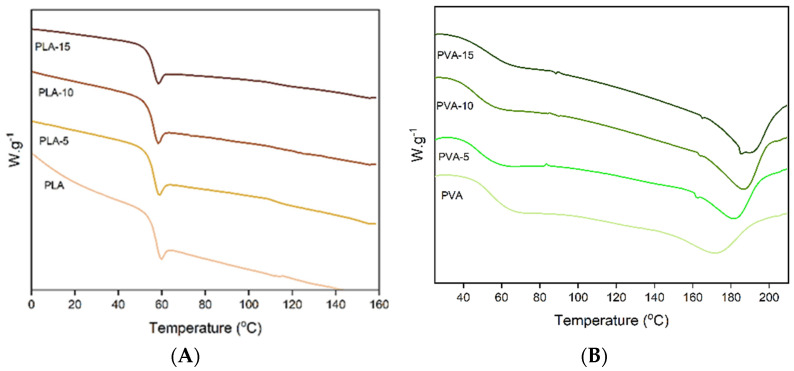
DSC thermograms (second heating step) obtained for PLA (**A**) and PVA (**B**) films (conditioned at 0% RH) with 0, 5, 10, and 15 wt.% of AS powder.

Thermal analysis was carried out to identify phase transitions in the polymers as well thermal stability. Figure 5 shows the DSC thermograms (second heating) of PLA and PVA films with different contents of AS powder. The glass transition of the amorphous PLA was observed, as well as the glass transition and melting endotherm of PVA in each kind of film. Table 5 summarises the values obtained for glass transition temperatures (Tg), melting temperatures (Tm) and melting enthalpies (ΔHm) of the different samples. The crystallisation temperatures (Tc) of PVA deduced from the cooling step were also included in Table 5. The Tg values of PLA were around 55 °C, similar to those reported by other authors for pure amorphous PLA [49,57]. These values were not significantly affected by the incorporation of AS powder, which suggests that no plasticising compounds were released from the AS particles, affecting the amorphous phase of the polymer. In contrast, the Tg values of the PVA films decreased as the AS powder rose in the composite (from 55 °C to 45 °C). This could be attributed to the increase in the glycerol–polymer ratio (Table 1) when the AS rose or to the release of plasticising compounds from the AS during the film thermoprocessing that contributed to promote the molecular mobility. The analysis of an additional sample maintaining the glycerol-polymer ratio of the PVA films, and the 15% of AS showed a slight increase in the Tg (59.5 ± 1.5) with respect to the sample PVA-15. Therefore, small differences in the available glycerol for the polymer greatly affected the polymer plasticisation. Moreover, glycerol could be partially bonded to the filler, resulting in overlapped effects on the polymer plasticisation.

**Figure 6:**
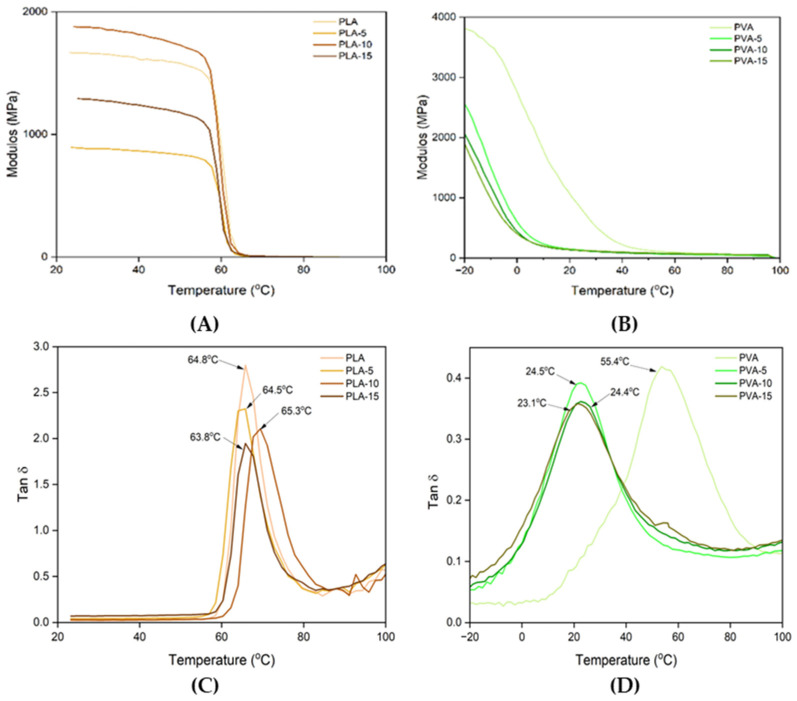
Storage modulus (**A**,**B**) and tan δ (**C**,**D**) as a function of temperature for PLA (**A**,**C**) and PVA (**B**,**D**) films with 0, 5, 10, and 15 wt.% AS powder, obtained from DMA.

The different effect of the AS filler on the glass transition of composites was also analysed through DMA for both PLA and PVA films. Figure 6 shows the thermograms (storage modulus and tan δ and vs. temperature) of the samples conditioned at 53% RH. In the PLA samples, the storage modulus of PLA was very high below 60 °C (1000–2000 MPa), which confirms that the materials remained in a glassy state. Above 60 °C, the modulus decreases significantly, signalling the onset of the glass transition, with the Tg close to 60 °C. Therefore, at room temperature, the material behaves as a glassy material [58]. The incorporation of AS powder did not significantly modify the Tg values of the films, as reported by other authors, when different fillers, such as glass fibres [59] and bleached cellulose fibres [60,61], were incorporated into the PLA matrix. Likewise, the Tg values found by DMA analysis were similar to those observed using the DSC for films conditioned at 0% RH, reflecting the low sensitivity of PLA to water plasticisation.

In contrast, the storage modulus of glycerol-plasticised PVA was higher than that of PLA and falls more progressively with the temperature increase, which led to a clear relaxation (peak of tan δ) of the polymer at 55 °C for neat PVA and at a lower temperature (23–24 °C) for composite films. The Tg value of neat PVA conditioned at 53% RH was very close to that obtained by DSC for film conditioned at 0% RH, despite the known plasticising effect of water in this polymer, and was lower than that reported for non-plasticised PVA (64 °C) [62]. This indicates that mechanical and calorimetric responses were not coherent for this polymer, as observed in other studies [63]. The incorporation of AS powder provoked an strong mechanical effect in the PVA matrix, highly reducing both the storage modulus and Tg values. However, other authors [63] reported an increase in the storage modulus, while the tan *δ* peak slightly shifted to lower temperatures, when micro-fibrillated cellulose was incorporated at different ratios into low-viscosity, fully hydrolysed PVA films without plasticiser, demonstrating a notable reinforcing effect of the filler. In contrast, the obtained behaviour of the AS filler suggests a strong weakening effect in the matrix, which could be explained by the release of polar compounds from AS to the polymer matrix and their interactions with the PVA chains. This may provoke a reduction in the interchain forces, as also deduced from the tensile properties, and a significant decrease in the Tg. Interactions of the filler and polymer with the glycerol could also contribute to the observed behaviour. In fact, an additional formulation maintaining the glycerol–polymer ratio of the PVA film, with 15% AS, exhibited a DMA Tg value of 49 °C, which was lower than but closer to that of PVA film. Therefore, the slight increase in the glycerol–polymer ratio in films containing AS fillers greatly affected the mechanical relaxation of the polymer matrix.

The PVA crystalline phase exhibited a wide melting endotherm (Figure 5) within the 150–200 °C temperature range, with peak temperature at 172 °C, as previously reported for this polymer [50]. When AS powder was incorporated, the peak temperature (Tm) and melting enthalpy (expressed per mass unit of polymer) tended to increase, while the crystallisation temperature rose. This suggests that AS particles promoted PVA crystallisation, giving rise to the formation of bigger crystals (higher Tm) with less supercooling requirements (higher Tc). Therefore, the AS particles had a nucleating effect in PVA composites, favouring PVA crystallisation and crystal growth, which can also be due to the observed plasticising effect that promote the chain mobility and crystallisation [64]. This effect was also observed for cellulose fibres obtained from almond shell when incorporated into PVA composites [50].

### 3.5. Thermal Stability of the Films

**Figure 7:**
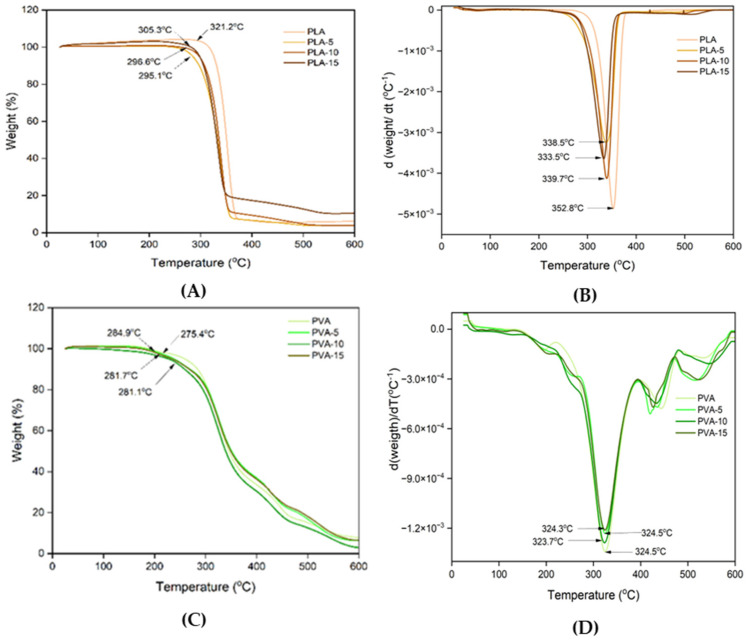
TGA (**A**,**C**) and DTGA (**B**,**D**) curves of PLA (**A**,**B**) and PVA (**C**,**D**) films with 0, 5, 10, and 15 wt.% AS powder.

Thermogravimetric analysis (TGA) was carried out in order to assess the influence of the filler on thermal stability of the polymers. Figure 7 shows the TGA and DGTA curves of the different films The PLA neat films exhibited the typical degradation described in previous studies [52], with onset and peak temperature (maximum degradation rate in DGTA curve) at 320 and 352 °C, respectively. The incorporation of AS particles at different ratios slightly shifted these temperatures to lower values (about 30 °C), which indicates that a partial hydrolysis of the chains was provoked during the film thermoprocessing due to the release of bound water from the AS filler. This hydrolytic effect in thermoprocessed PLA matrices was also observed for other lignocellulosic fillers such as those obtained from Posidonia oceanica waste [65] and other cellulosic materials [66].

For the PVA films, a slight weight loss was observed around 160–200 °C, due to the degradation/vaporisation of glycerol, as observed by other authors in glycerol plasticised PVA films [11,50]. The degradation of PVA occurred between 275–370 °C, with peak temperature at 325 °C, as previously reported for this polymer [50]. The incorporation of AS powder in different proportions did not lead to any significant shift in the polymer degradation temperatures, while it provoked slight modifications in the curves due to the overlapped degradation of the relatively low amount of AS components, which occurs in a wide temperature range [23], in line with the AS complex composition (mainly proteins, cellulose, hemicellulose, and lignin). Similar behaviour was observed for PVA composites with other cellulosic materials [67].

### 3.6. Antioxidant Properties of Films

The antioxidant capacity of the films was evaluated through their ability to scavenge the ABTS^+^ radical in liquid media and by their capacity to preserve sunflower oil from oxidation when packaged in single-dose bags of the different materials.

**Figure 8:**
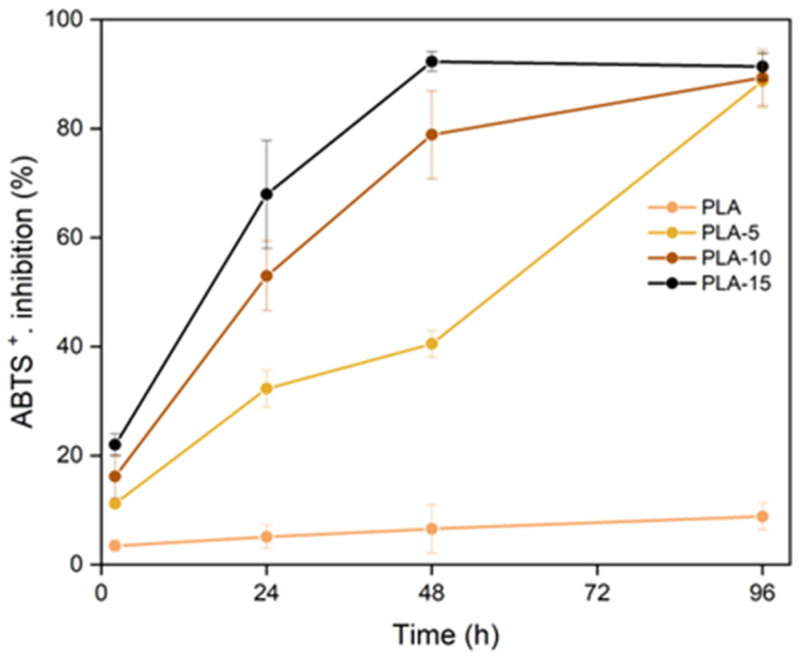
Inhibition of the ABTS^+^ radical by the different PLA films with 0, 5, 10, and 15 wt.% of almond skin powder.

The antioxidant capacity of bioactive substances often depends on the neutralisation of radicals, such as DPPH or ABTS^+^, since oxidative processes are generally based on complex reactions mediated by the production of free radicals [68]. The ABTS^+^ scavenging test confirms the antioxidant properties of the phenolic compounds present in the AS particles of composites. Figure 8 shows the percentage inhibition of radical by PLA films with different AS ratios, as a function of contact time with the liquid medium, where a clear dependence on the AS ratio was observed. The inhibition increased as the exposure time rose, in line with the progressive release of antioxidant compounds into the oxidative medium. Almond skin is rich in phenolic acids (mainly protocatechuic, chlorogenic, vanillic, and sinapic acids) and anthocyanidins and procyanidins (mainly catechin and epicatechin), all these with high antioxidant power [69]. The greater the AS ratio and the antioxidant components, the higher the inhibition capacity of the films, while for pure PLA film, a minimum inhibition was observed over time.

Due the high solubility of PVA in the liquid medium, the release of antioxidants occurred very fast (about 30 min) when the maximum inhibition percentage (78%) was observed for every sample, regardless of the AS content, while the PVA films without AS had % inhibition near zero. This suggests that the radical scavengers were less protected during the film thermoprocessing and storage in this polar matrix and only a practically constant activity remained in the different films.

**Figure 9:**
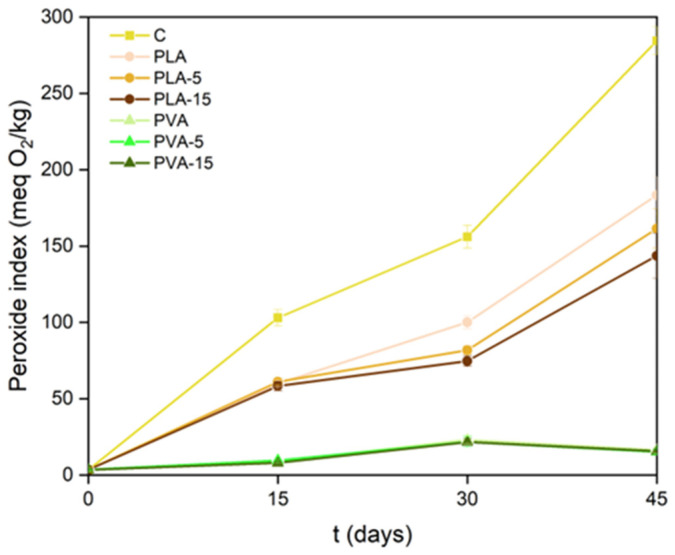
Development of peroxide index of sunflower oil packed in single-dose bags of different PLA and PVA films with 0, 5, and 15 wt.% of AS powder throughout storage time.

**Figure 10:**
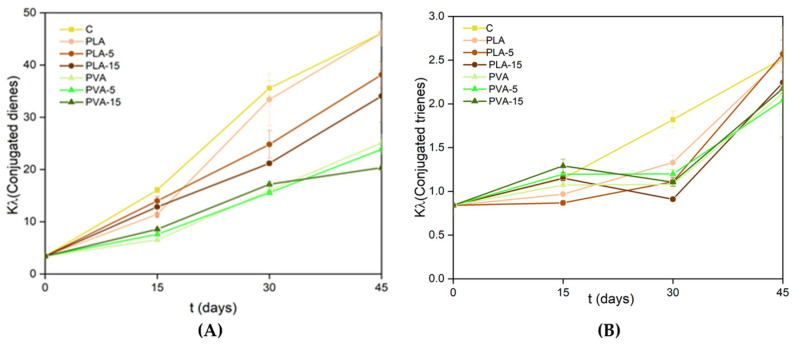
Values of the extinction coefficients of conjugated dienes (k_232_) (**A**) and trienes (k_268_) (**B**) at different storage times of sunflower oil packaged in single-dose bags of different PLA and PVA composite films with 0, 10, and 15 wt.% of AS powder.

The antioxidant efficacy of the films was also evaluated based on their capability to inhibit the oxidation of packaged sunflower oil. The ability will be affected by the oxygen permeability of the films, which controls the oxygen access to the oil samples, their UV-light-blocking effect that protects from light-induced oxidation reaction, and the potential antioxidant properties of the compounds from AS powder that may be released into the oil. The oxidation degree of the different oil samples was analysed through the peroxide index and the formation of conjugated dienes and trienes, as established by EU standard 2568/91 [40]. The values obtained at different storage times are shown in Figure 9 and Figure 10 for the oil samples packed in the different PLA and PVA films with 0, 5%, and 15% AS powder, respectively. To evaluate the effectiveness of the oxidative test, an open control sample (C) was also analysed at different times. This control sample showed higher values of PI and dienes and trienes than the packaged oil samples due to the oxygen barrier of the materials, which limited the access of oxygen to the sample.

Comparing the samples packaged in both materials, the low level of oxidation of the oil packaged in PVA bags stands out, mainly due to its high oxygen barrier capacity. In these samples, no significant effect of the AS powder in the material on the oil oxidation levels was detected. In contrast, the effect of AS antioxidants was evident in samples packaged in PLA films with lower oxygen barrier capacity, which was significantly improved in composite films. For the 15% of AS in the PLA matrix, the highest delay in the oxidative process of the oil was observed in terms of PI and dienes. Conjugated dienes, with absorbance maximum at 232 nm, are formed by the oxidation of fatty acids due to the displacement of the double bond of unsaturated fatty acids during the peroxide formation [70]. Therefore, a correlation between the PI values and diene concentrations exists since both are the result of primary oxidation [71]. Trienes are formed because of secondary oxidation and have their maximum absorbance at 270 nm. Therefore, measurement of the absorbance of the oil at 232 and 270 nm allows for estimation of its oxidative state. The concentration of trienes did not change noticeably until 45 days of storage in packaged oil samples (Figure 10), when secondary oxidation compounds were formed. The oil packed in PVA films showed the lowest formation of trienes at 45 storage days, together with the oil packed in PLA with 15% almond powder. This indicates the protective effect of the antioxidants potentially released into the oil since, although the 15% AS particles in PLA films decrease their oxygen permeability by 22%, the values of this property were about 15 times greater than those of PVA with different proportions of AS. With the low values of oxygen permeability of PVA films, the antioxidant effect of AS phenolics was not appreciated.

## 4. Conclusions

The incorporation of up to 15% of almond skin powder into PLA and PVA polymer matrices was possible while maintaining the mechanical performance of the films relatively close to the corresponding neat polymer, despite the weakening effect on the PVA matrix and the small alterations in its crystallization pattern. The particles provided the films with the characteristic colour of the powder and strong UV light-blocking capacity, while improving the oxygen barrier capacity of both type of polymeric matrices (24% in PLA with 15% AS and 42% in PVA with 10% AS). The water vapour permeability increased in PLA films (by 192% with 15% AS) but decreased in PVA films, especially with low AS content (by 19% with 5% AS).

AS particles provided the PLA and PVA films with antioxidant properties due to the phenolic compounds of the filler, improving the oxygen barrier capacity and delaying the unsaturated oil oxidation. This was reflected in the lower values of the peroxide index, and conjugates dienes and trienes of the sunflower oil packaged in single-dose bags of the different materials. For the PVA films, the high oxygen barrier capacity of the bags was the key factor for the oil preservation, masking the antioxidant capacity of the AS particles.

These composites represent an interesting alternative for the valorisation of AS, reducing the overall cost of packaging materials while providing them with additional functions. Nevertheless, further studies are needed to validate these composites as antioxidant packaging materials in other oxidation-prone food matrices.

## Figures and Tables

**Table 1:** Mass fraction of different components in the PVA and PLA composite films, with 0%, 5%, 10%, and 15% of AS powder.

Sample	PLA	PVA	Gly	AS
**PLA**	1.00	-	-	0.00
**PLA-5**	0.95	-	-	0.05
**PLA-10**	0.90	-	-	0.10
**PLA-15**	0.85	-	-	0.15
**PVA**	-	0.90	0.10	0.00
**PVA-5**	-	0.85	0.10	0.05
**PVA-10**	-	0.80	0.10	0.10
**PVA-15**	-	0.75	0.10	0.15

**Table 2:** Colour coordinates (L*, Cab*, and hue*) of the different PLA and PVA composite films with 0, 5, 10, and 15 wt.% ground almond skin, and the total colour differences with respect to the corresponding film without AS particles.

Sample		L*	C_ab_*	h_ue_*	ΔE
**PLA**	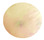	75.5 ± 0.8 ^a1^	35.0 ± 9.0 ^b1^	56.0 ± 6.0 ^a1^	-
**PLA-5**	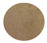	37.0 ± 4.0 ^b1^	99.0 ± 8.0 ^a1^	53.7 ± 0.9 ^ab1^	42 ± 3 ^a1^
**PLA-10**	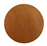	31.5 ± 1.7 ^b1^	97.5 ± 1.7 ^a1^	48.4 ± 1.3 ^ab1^	44 ± 1 ^a1^
**PLA-15**	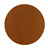	31.0 ± 2.0 ^b1^	100.0 ± 20.0 ^a1^	46.2 ± 1.5 ^b1^	45 ± 0 ^a1^
**PVA**	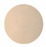	73.2 ± 0.9 ^a2^	16.0 ± 3.0 ^c2^	46.0 ± 11.0 ^a2^	-
**PVA-5**	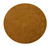	29.5 ± 0.1 ^b2^	45.0 ± 2.0 ^a2^	49.3 ± 0.1 ^a2^	44 ± 1 ^b2^
**PVA-10**	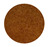	26.8 ± 0.2 ^c2^	29.3 ± 0.2 ^b2^	40.0 ± 0.7 ^a2^	46 ± 1 ^ab2^
**PVA-15**	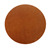	26.7 ± 0.1 ^c2^	30.3 ± 0.8 ^b2^	37.0 ± 0.5 ^a2^	47 ± 1 ^a2^

Different letters a,b,c in the same column indicates significant differences between formulations (*p* < 0.05). Different numbers 1,2 in the same column indicate a significant difference between formulations with a determined AS ratio (*p* < 0.05).

**Table 3:** Tensile parameters (elastic modulus: EM, tensile strength: TS, and deformation at break: E%) and permeability to water vapour (WVP) and oxygen (OP) of different PLA and PVA films with 0, 5, 10, and 15 wt.% of almond skin powder.

Sample	OP × 10^14^ (cm^3^/m s Pa)	WVP × 10^11^ (g/Pa s m)	EM (MPa)	TS (MPa)	E (%)
PLA	196.0 ± 5.0 ^a1^	4.9 ± 1.1 ^c1^	1360 ± 60 ^ab1^	49.0 ± 2.0 ^a1^	5.0 ± 0.4 ^a1^
PLA-5	175.0 ± 4.0 ^b1^	9.8 ± 0.4 ^b1^	1400 ± 90 ^a1^	43.0 ± 3.0 ^ab1^	3.3 ± 0.2 ^b1^
PLA-10	164.0 ± 5.0 ^bc1^	12.6 ± 0.5 ^ab1^	1340 ± 40 ^ab1^	35.4 ± 1.1 ^ab1^	3.4 ± 0.6 ^ab1^
PLA-15	149.0 ± 5.0 ^c1^	14.3 ± 1.3 ^a1^	1300 ± 70 ^b1^	32.4 ± 0.9 ^b1^	3.4 ± 0.4 ^ab1^
PVA	11.5 ± 0.8 ^a2^	219.0 ± 5.0 ^a2^	123 ± 7 ^a2^	44.0 ± 7.0 ^a2^	85.1 ± 0.6 ^a2^
PVA-5	8.1 ± 1.6 ^bc2^	178.0 ± 5.0 ^c2^	85 ± 9 ^b2^	33.0 ± 3.0 ^ab2^	84.6 ± 0.9 ^a2^
PVA-10	6.7 ± 0.7 ^c2^	190.7 ± 0.5 ^bc2^	95 ± 11 ^b2^	30.0 ± 3.0 ^ab2^	85.0 ± 4.0 ^a2^
PVA-15	9.5 ± 0.1 ^ab2^	202.0 ± 8.0 ^ab2^	103 ± 20 ^b2^	21.0 ± 4.0 ^b2^	58.0 ± 11.0 ^b2^

Different letters a,b,c in the same column indicate a significant difference between formulations of a determined polymer (*p* < 0.05). Different numbers 1,2 in the same column indicate a significant difference between formulations with a determined AS ratio (*p* < 0.05).

**Table 4:** Thickness, equilibrium moisture content at 53% relative humidity, and water contact angle of different PLA and PVA films with 0, 5, 10, and 15 wt.% of almond skin powder.

Sample	Thickness (mm)	Equilibrium Moisture Content (53% HR)	Contact Angle (°)
PLA	0.14 ± 0.00 ^b1^	0.2 ± 0.1 ^a1^	92 ± 8 ^a1^
PLA-5	0.15 ± 0.01 ^a1^	0.3 ± 0.1 ^a1^	68 ± 9 ^b1^
PLA-10	0.14 ± 0.01 ^b1^	0.4 ± 0.0 ^a1^	64 ± 6 ^b1^
PLA-15	0.14 ± 0.00 ^b1^	0.4 ± 0.1 ^a1^	64 ± 6 ^b1^
PVA	0.17 ± 0.00 ^c2^	12.1 ± 1.1 ^a2^	44 ± 11 ^a2^
PVA-5	0.18 ± 0.02 ^b2^	10.1 ± 1.4 ^a2^	35 ± 8 ^b2^
PVA-10	0.19 ± 0.03 ^a2^	9.0 ± 3.0 ^a2^	38 ± 7 ^b2^
PVA-15	0.18 ± 0.00 ^b2^	8.9 ± 0.5 ^a2^	39 ± 7 ^b2^

Different letters a,b,c in the same column indicate a significant difference between formulations of a determined polymer (*p* < 0.05). Different numbers 1,2 in the same column indicate a significant difference between formulations with a determined AS ratio (*p* < 0.05).

**Table 5:** Glass transition, crystallisation temperatures, melting temperatures, and enthalpy of fusion of PVA in films with 0, 5, 10, and 15 wt.% AS powder.

Sample	Tg (°C)	Sample	Tg (°C)	Tc (°C)	Tm (°C)	ΔHm (J/g)
**PLA**	56.1 ± 0.0 ^a^	**PVA**	55.2 ± 1.6 ^a^	129.2 ± 0.5 ^d^	172.1 ± 0.7 ^c^	23.0 ± 0.3 ^b^
**PLA-5**	55.1 ± 0.2 ^a^	**PVA-5**	50.3 ± 0.8 ^ab^	144.1 ± 0.9 ^c^	182.2 ± 0.9 ^b^	27.5 ± 1.0 ^b^
**PLA-10**	55.3 ± 0.7 ^a^	**PVA-10**	46.1 ± 2.1 ^bc^	152.9 ± 0.3 ^b^	186.1 ± 0.4 ^a^	35.2 ± 2.7 ^a^
**PLA-15**	54.9 ± 0.3 ^a^	**PVA-15**	44.7 ± 1.0 ^c^	159.4 ± 1.3 ^a^	185.8 ± 1.1 ^a^	37.5 ± 2.0 ^a^

Different letters a, b, c, d in the same column indicate a significant difference between formulations (*p* < 0.05).

## Data Availability

The original contributions presented in the study are included in the article, further inquiries can be directed to the corresponding author.

## References

[1] Topuz F., Uyar T. (2020). Antioxidant, antibacterial and antifungal electrospun nanofibers for food packaging applications. Food Res. Int..

[2] Turan D., Keukens B.M., Schifferstein H.N. (2024). Food packaging technology considerations for designers: Attending to food, consumer, manufacturer, and environmental issues. Compr. Rev. Food Sci. Food Saf..

[3] Operato L., Panzeri A., Masoero G., Gallo A., Gomes L., Hamd W. (2025). Food packaging use and post-consumer plastic waste management: A comprehensive review. Front. Food Sci. Technol..

[4] Hemavathi A.B., Siddaramaiah H. (2018). Food packaging: Polymers as packaging materials in food supply chains. Encycl. Polym. Appl..

[5] Rillig M.C., Kim S.W., Kim T.Y., Waldman W.R. (2011). The global plastic toxicity debt. Environ. Sci. Technol..

[6] Yao Z., Seong H.J., Jang Y. (2022). Environmental toxicity and decomposition of polyethylene. Ecotoxicol. Environ. Saf..

[7] Nilsen-Nygaard J., Fernández E.N., Radusin T., Rotabakk B.T., Sarfraz J., Sharmin N., Sivertsvik M., Sone I., Pettersen M.K. (2021). Current status of biobased and biodegradable food packaging materials: Impact on food quality and effect of innovative processing technologies. Compr. Rev. Food Sci. Food Saf..

[8] Weligama Thuppahige V.T., Karim M.A. (2022). A comprehensive review on the properties and functionalities of biodegradable and semibiodegradable food packaging materials. Compr. Rev. Food Sci. Food Saf..

[9] Thulasisingh A., Kumar K., Yamunadevi B., Poojitha N., SuhailMadharHanif S., Kannaiyan S. (2022). Biodegradable packaging materials. Polym. Bull.

[10] Andrade J., González-Martínez C., Chiralt A. (2020). The incorporation of carvacrol into poly (vinyl alcohol) films encapsulated in lecithin liposomes. Polymers.

[11] Andrade J., González-Martínez C., Chiralt A. (2022). Antimicrobial PLA-PVA multilayer films containing phenolic compounds. Food Chem..

[12] Mohan S., Panneerselvam K. (2020). A short review on mechanical and barrier properties of polylactic acid-based films. Mater. Today Proc..

[13] Da Silva Pens C.J., Klug T.V., Stoll L., Izidoro F., Flores S.H., de Oliveira Rios A. (2024). Poly (lactic acid) and its improved properties by some modifications for food packaging applications: A review. Food Packag. Shelf Life.

[14] Mangaraj S., Thakur R.R., Yadav A. (2022). Development and characterization of PLA and Cassava starch-based novel biodegradable film used for food packaging application. J. Food Process. Preserv..

[15] Cinelli P., Seggiani M., Coltelli M.B., Danti S., Righetti M.C., Gigante V., Sandroni M., Signori F., Lazzeri A. (2021). Overview of agro-food waste and by-products valorization for polymer synthesis and modification for bio-composite production. Proceedings.

[16] Priyadarshi R., Ghosh T., Purohit S.D., Prasannavenkadesan V., Rhim J.W. (2024). Lignin as a sustainable and functional material for active food packaging applications: A review. J. Clean. Prod..

[17] Rojas A., Velásquez E., Patiño Vidal C., Guarda A., Galotto M.J., López de Dicastillo C. (2021). Active PLA packaging films: Effect of processing and the addition of natural antimicrobials and antioxidants on physical properties, release kinetics, and compostability. Antioxidants.

[18] Jiang Y., Zhang Y., Deng Y. (2023). Latest advances in active materials for food packaging and their application. Foods.

[19] Ganeson K., Mouriya G.K., Bhubalan K., Razifah M.R., Jasmine R., Sowmiya S., Amirul A.A., Vigneswari S., Ramakrishna S. (2023). Smart packaging—A pragmatic solution to approach sustainable food waste management. Food Packag. Shelf Life.

[20] Angellier-Coussy H., Gastaldi E., Gontard N., Guillaume C., Guillard V., Peyron S. (2024). Converting Agro-industrial By-products into Biodegradable Composite Materials for Food Packaging: Presentation of an Eco-reasoned Approach. Green Chemistry and Agro-Food Industry: Towards a Sustainable Bioeconomy.

[21] Varghese S.A., Pulikkalparambil H., Promhuad K., Srisa A., Laorenza Y., Jarupan L., Nampitch T., Chonhenchob V., Harnkarnsujarit N. (2023). Renovation of agro-waste for sustainable food packaging: A review. Polymers.

[22] INC International Nut and Dried Fruit Council (2023). Nut and Dried Fruit Statistical Yearbook 2022–23.

[23] Freitas P.A., Martín-Pérez L., Gil-Guillén I., González-Martínez C., Chiralt A. (2023). Subcritical water extraction for valorisation of almond skin from almond industrial processing. Foods.

[24] Prgomet I., Gonçalves B., Domínguez-Perles R., Pascual-Seva N., Barros A.I. (2017). Valorization challenges to almond residues: Phytochemical composition and functional application. Molecules.

[25] Smeriglio A., Mandalari G., Bisignano C., Filocamo A., Barreca D., Bellocco E., Trombetta D. (2016). Polyphenolic content and biological properties of Avola almond (Prunus dulcis Mill. DA Webb) skin and its industrial byproducts. Ind. Crops Prod..

[26] Alalwan T.A., Mohammed D., Hasan M., Sergi D., Ferraris C., Gasparri C., Rondanlli M., Perna S. (2022). Almond, hazelnut, and pistachio skin: An opportunity for nutraceuticals. Nutraceuticals.

[27] Özcan M.M. (2023). A review on some properties of almond: Impact of processing, fatty acids, polyphenols, nutrients, bioactive properties, and health aspects. J. Food Sci. Technol.

[28] Ordoñez R., Atarés L., Chiralt A. (2023). Multilayer antimicrobial films based on starch and PLA with superficially incorporated ferulic or cinnamic acids for active food packaging purposes. Food Chem. Adv..

[29] Timón M., Andrés A.I., Sorrentino L., Cardenia V., Petrón M.J. (2022). Effect of phenolic compounds from almond skins obtained by water extraction on pork patty shelf life. Antioxidants.

[30] García A.V., Santonja M.R., Sanahuja A.B., Selva M.D.C.G. (2014). Characterization and degradation characteristics of poly (ε-caprolactone)-based composites reinforced with almond skin residues. Polym. Degrad. Stab..

[31] Valdés A., Dominici F., Fortunati E., Kenny J.M., Jiménez A., Garrigós M.C. (2023). Effect of almond skin waste and glycidyl methacrylate on mechanical and color properties of poly (ε-caprolactone)/poly (lactic acid) blends. Polymers.

[32] Singh R., Kumar R., Pawanpreet, Singh M., Singh J. (2022). On mechanical, thermal and morphological investigations of almond skin powder-reinforced polylactic acid feedstock filament. J. Thermoplast. Compos. Mater..

[33] Singh M., Kumar S., Singh R., Kumar R., Kumar V. (2022). On shear resistance of almond skin reinforced PLA composite matrix-based scaffold using cancellous screw. Adv. Mater. Process. Technol..

[34] Mankotia K., Singh I., Singh R. (2020). On effect of almond skin powder waste reinforcement in PA6: Rheological, thermal and wear properties. Mater. Today Proc..

[35] (2005). Standard Test Methods for Water Vapor Transmission of Materials.

[36] (2010). Standard Test Method for Oxygen Gas Transmission Rate Through Plastic Film and Sheeting Using s Coulometric Sensor.

[37] (2012). Standard Test Method for Tensile Properties of Thin Plastic Sheeting.

[38] Peixoto A.M., Petronilho S., Domingues M.R., Nunes F.M., Lopes J., Pettersen M.K., Grøvlen M.S., Wetterhus E.M., Gonçalves I., Coimbra M.A. (2023). Potato chips byproducts as feedstocks for developing active starch-based films with potential for cheese packaging. Foods.

[39] Nunes C., Maricato É., Cunha Â., Nunes A., da Silva J.A.L., Coimbra M.A. (2013). Chitosan–caffeic acid–genipin films presenting enhanced antioxidant activity and stability in acidic media. Carbohydr. Polym..

[40] Commission Regulation (EEC) No 2568/91 (1991). Relativo a las características de los aceites de oliva y de los aceites de orujo de oliva y sobre sus métodos de análisis. D. Of. L.

[41] Wang Q., Ji C., Sun J., Zhu Q., Liu J. (2020). Structure and properties of polylactic acid biocomposite films reinforced with cellulose nanofibrils. Molecules.

[42] Popa E.E., Rapa M., Popa O., Mustatea G., Popa V.I., Mitelut A.C., Popa M.E. (2017). Polylactic acid/cellulose fibres based composites for food packaging applications. Mater. Plast.

[43] Darie-Niţă R.N., Vasile C., Stoleru E., Pamfil D., Zaharescu T., Tarţău L., Tudorachi N., Brebu M.A., Pricope G.M., Dumitriu R.P. (2018). Evaluation of the rosemary extract effect on the properties of polylactic acid-based materials. Materials.

[44] Karagöz İ. (2024). Production and characterization of sustainable biocompatible PLA/walnut shell composite materials. Polym. Bull..

[45] Franca T., Goncalves D., Cena C. (2022). ATR-FTIR spectroscopy combined with machine learning for classification of PVA/PVP blends in low concentration. Vibr. Spectrosc..

[46] Barbălată-Mândru M., Serbezeanu D., Butnaru M., Rîmbu C.M., Enache A.A., Aflori M. (2022). Poly (vinyl alcohol)/plant extracts films: Preparation, surface characterization and antibacterial studies against gram positive and gram negative bacteria. Materials.

[47] Kashid S.M., Bagchi S. (2014). Experimental determination of the electrostatic nature of carbonyl hydrogen-bonding interactions using IR-NMR correlations. J. Phys. Chem. Lett..

[48] Yu H., Qin Z., Yan C., Yao J. (2014). Green nanocomposites based on functionalized cellulose nanocrystals: A study on the relationship between interfacial interaction and property enhancement. ACS Sustain. Chem. Eng..

[49] Martin-Perez L., Contreras C., Chiralt A., Gonzalez-Martinez C. (2025). Active Polylactic Acid (PLA) Films Incorporating Almond Peel Extracts for Food Preservation. Molecules.

[50] Gil-Guillén I., González-Martínez C., Chiralt A. (2025). Influence of the Cellulose Purification Method on the Properties of PVA Composites with Almond Shell Fibres. Molecules.

[51] Limpan N., Prodpran T., Benjakul S., Prasarpran S. (2012). Influences of degree of hydrolysis and molecular weight of poly (vinyl alcohol) (PVA) on properties of fish myofibrillar protein/PVA blend films. Food Hydrocoll..

[52] Ordoñez R., Atarés L., Chiralt A. (2022). Effect of ferulic and cinnamic acids on the functional and antimicrobial properties in thermo-processed PLA films. Food Packag. Shelf Life.

[53] Li L., Xu X., Liu L., Song P., Cao Q., Xu Z., Fang Z., Wang H. (2020). Water governs the mechanical properties of poly (vinyl alcohol). Polymer.

[54] Vengadesan E., Morakul S., Muralidharan S., Pullela P.K., Alarifi A., Arunkumar T. (2025). Enhancement of polylactic acid (PLA) with hybrid biomass-derived rice husk and biocarbon fillers: A comprehensive experimental study. Discov. Appl. Sci..

[55] Li L., Ding S., Zhou C. (2004). Preparation and degradation of PLA/chitosan composite materials. J. Appl. Polym. Sci..

[56] Freitas P.A., Gil N.J.B., González-Martínez C., Chiralt A. (2022). Antioxidant poly (lactic acid) films with rice straw extract for food packaging applications. Food Packag. Shelf Life.

[57] Greco A., Ferrari F. (2021). Thermal behavior of PLA plasticized by commercial and cardanol-derived plasticizers and the effect on the mechanical properties. J. Therm. Anal. Calorim..

[58] Cristea M., Ionita D., Iftime M.M. (2020). Dynamic mechanical analysis investigations of PLA-based renewable materials: How are they useful?. Materials.

[59] Akindoyo J.O., Beg M.D.H., Ghazali S., Heim H.P., Feldmann M., Mariatti M. (2021). Simultaneous impact modified and chain extended glass fiber reinforced poly (lactic acid) composites: Mechanical, thermal, crystallization, and dynamic mechanical performance. J. Appl. Polym. Sci..

[60] Espinach F.X., Boufi S., Delgado-Aguilar M., Julián F., Mutjé P., Méndez J.A. (2018). Composites from poly (lactic acid) and bleached chemical fibres: Thermal properties. Compos. B. Eng..

[61] Frone A.N., Berlioz S., Chailan J.F., Panaitescu D.M., Donescu D.J.P.C. (2011). Cellulose fiber-reinforced polylactic acid. Polym. Comp..

[62] Li N., Xiao C., An S., Hu X. (2010). Preparation and properties of PVDF/PVA hollow fiber membranes. Desalination.

[63] Lu J., Wang T., Drzal L.T. (2008). Preparation and properties of microfibrillated cellulose polyvinyl alcohol composite materials. Compos. Part A Appl. Sci. Manuf..

[64] Thomas D., Cebe P. (2017). Self-nucleation and crystallization of polyvinyl alcohol. J. Therm. Anal. Calorim..

[65] Camarena-Bononad P., Freitas P.A., González-Martínez C., Chiralt A., Vargas M. (2024). Influence of the Purification Degree of Cellulose from Posidonia oceanica on the Properties of Cellulose-PLA Composites. Polysaccharides.

[66] Mokhena T.C., Sefadi J.S., Sadiku E.R., John M.J., Mochane M.J., Mtibe A. (2018). Thermoplastic processing of PLA/cellulose nanomaterials composites. Polymers.

[67] Rowe A.A., Tajvidi M., Gardner D.J. (2016). Thermal stability of cellulose nanomaterials and their composites with polyvinyl alcohol (PVA). J. Therm. Anal. Calorim.

[68] Chaves N., Santiago A., Alías J.C. (2020). Quantification of the antioxidant activity of plant extracts: Analysis of sensitivity and hierarchization based on the method used. Antioxidants.

[69] Esfahlan A.J., Jamei R., Esfahlan R.J. (2010). The importance of almond (*Prunus amygdalus* L.) and its by-products. Food Chem..

[70] Shahidi F., Zhong Y. (2015). Measurement of antioxidant activity. J. Funct. Foods.

[71] Srinivasan S., Xiong Y.L., Decker E.A. (1996). Inhibition of protein and lipid oxidation in beef heart surimi-like material by antioxidants and combinations of pH, NaCl, and buffer type in the washing media. J. Agric. Food Chem..

